# The Role of Macrophages in the Immune Response to Peritoneal Infection with *Dirofilaria immitis* Larvae in the Mongolian Gerbil (*Meriones unguiculatus*)

**DOI:** 10.3390/pathogens15080851

**Published:** 2026-08-14

**Authors:** Elyssa Campbell, Catherine Pope, Katelin Greenway, Bridget Garner, Kaori Sakamoto, Andrew Moorhead

**Affiliations:** 1 Department of Infectious Diseases, College of Veterinary Medicine, University of Georgia, Athens, GA 30602, USA; 2 Division of Animal Resources, Emory University, Atlanta, GA 30322, USA; 3 Veterinary Medicine Research and Development, Zoetis, Kalamazoo, MI 49007, USA; 4 Department of Pathology, College of Veterinary Medicine, University of Georgia, Athens, GA 30602, USA; 5 Department of Population Health and Pathobiology, College of Veterinary Medicine, North Carolina State University, Raleigh, NC 27607, USA; 6 Department of Clinical Sciences, College of Veterinary Medicine, North Carolina State University, Raleigh, NC 27607, USA

**Keywords:** *Dirofilaria immitis*, dirofilariasis, macrophages, Mongolian gerbil, innate immunity, neutrophils, host specificity, clodronate liposomes, flow cytometry, peritoneal cavity

## Abstract

The host cellular mechanisms determining whether *Dirofilaria immitis* (canine heartworm) larvae establish infection remain poorly understood. The Mongolian gerbil (*Meriones unguiculatus*), a naturally nonpermissive host, mounts a rapid macrophage-dominated peritoneal cellular response to intraperitoneal (IP) larval challenge. To test whether macrophages mediate larval clearance, peritoneal macrophages were selectively depleted with IP clodronate liposomes (100 µL/10 g body weight; days −4 and −1 relative to infection) before challenge with approximately 40 *D. immitis* third-stage larvae (L3). A rat anti-mouse anti-F4/80 antibody was validated as a cross-reactive macrophage marker in jird peritoneal exudate cells (PECs) by standard and imaging flow cytometry, and depletion efficacy was confirmed prior to infection. Significantly more L3 were recovered from macrophage-depleted jirds (mean = 17.7%; SEM = 6.0%) than from PBS liposome controls (mean = 3.8%; SEM = 1.9%) at 1-day post-infection (*p* < 0.03). Cytology and flow cytometry confirmed near-complete macrophage depletion, and a significant compensatory neutrophilia was observed in depleted animals (64.5% ± 5.7% vs. 19.6% ± 5.1% in controls; *p* < 0.0001). These findings establish peritoneal macrophages as the primary innate cellular mediators of *D. immitis* L3 clearance in a nonpermissive host, providing a validated in vivo framework for mechanistic dissection of filarial host specificity.

## 1. Introduction

The causative agent of canine heartworm disease, *Dirofilaria immitis*, is a mosquito-transmitted filarial nematode whose adult stage occupies the cardiac chambers and pulmonary vasculature of susceptible definitive hosts [1,2,3]. Transmission occurs during the blood meal of an infected mosquito, which deposits infective third-stage larvae (L3) onto host skin; these larvae subsequently undergo an extensive tissue migration lasting approximately six months before reaching the pulmonary arteries as sexually mature adults [4]. Clinically, heartworm disease in dogs is divided into severity classes (I-IV) by the American Heartworm Society, ranging from asymptomatic infection (Class I) to pronounced exercise intolerance with radiographic and echocardiographic abnormalities (Classes II–III) and life-threatening right-heart failure with caval syndrome (Class IV) [5]. Given the severe cardiopulmonary morbidity and mortality associated with adult worm burden, both the American Heartworm Society and the Companion Animal Parasite Council recommend continuous year-round chemoprophylaxis for dogs and cats in endemic regions [1,5,6].

The vertebrate immune system mounts two functionally distinct categories of response upon pathogen encounter: innate immunity and adaptive immunity [7]. Innate immunity constitutes the initial line of defense, engaging rapidly through pattern-recognition mechanisms that enable immune effector cells, including macrophages, neutrophils, mast cells, and dendritic cells, to identify and neutralize invaders without prior antigen-specific sensitization [7]. Adaptive immunity, by contrast, develops on a slower timescale and confers pathogen-specific protection that is amplified upon repeated exposure.

Among the effector cells of innate immunity, macrophages occupy a particularly central and functionally versatile role. Exposure to helminths characteristically drives a T helper type 2 (Th2) cytokine environment that polarizes tissue macrophages toward alternative activation [8,9,10]. Alternatively activated macrophages (AAMs) have been documented as active participants in host immune responses to filarial nematodes [11,12], though the precise mechanistic contributions of AAMs to larval fate, whether promoting clearance, constraining inflammation, or facilitating parasite tolerance, remain incompletely resolved.

Parasite establishment within a given host reflects the combined influence of a broad array of host susceptibility variables, including microbiome composition, inoculum size, host sex, ambient temperature, environmental conditions, age, prior immune history, nutritional status, and genetic background [13]. Beyond these individual-level factors, the probability of successful larval establishment is also shaped by the phylogenetic relatedness and geographic co-occurrence of host and parasite species [14,15,16]. The definitive host range of *D. immitis* is encompasses members of the mammalian order Carnivora, though host competence varies considerably: canids serve as primary, fully permissive definitive hosts, whereas cats and ferrets function as imperfect hosts in which only partial larval development occurs, and many Carnivoran species are resistant to parasite establishment [17,18,19]. The geographic distribution of *D. immitis* broadly corresponds to the range of competent *Culicidae* vector species across the Americas and other regions of endemicity [20,21].

The Mongolian gerbil (*Meriones unguiculatus*), commonly referred to as the jird, does not support the establishment of *D. immitis* infection [22], a finding consistent with its phylogenetic placement within the family Gerbillinae, order Rodentia [23,24], a lineage phylogenetically distant from the Carnivoran hosts that constitute the parasite’s natural range. Prior work by Evans et al. [25] established that intraperitoneal (IP) inoculation of jirds with *D. immitis* L3 provokes a rapid and parasite-dependent cellular mobilization in the peritoneal cavity: total peritoneal exudate cell (PEC) counts rise markedly by 1 dpi, and macrophages, comprising more than 40% of recovered PECs, were observed in direct physical contact with larvae. This cellular association implied that macrophages either mediate larval elimination directly or facilitate larval translocation from the peritoneal cavity into adjacent tissue compartments, thereby preventing larval persistence in the free peritoneal space. Collectively, these observations supported the hypothesis that peritoneal macrophages constitute the primary cellular mediators of the innate immune response to IP *D. immitis* L3 infection in this nonpermissive host [22,25,26].

## 2. Materials and Methods

### 2.1. Animals and Ethics Statement

Adult male Mongolian gerbils (*Meriones unguiculatus*) were obtained from a commercial supplier (Charles River Laboratories, Kingston, NY, USA) and group-housed under standard vivarium conditions [23] with ad libitum access to water and commercially available rodent diet. Purpose-bred dogs maintained at the University of Georgia served as the source of microfilaremic blood for parasite propagation. All experimental subjects were clinically healthy at enrollment and had no history of prior experimental procedures. All animal care and experimental procedures were conducted in full compliance with the guidelines and regulations of the University of Georgia Institutional Animal Care and Use Committee (IACUC), under approved protocol A2022 04-009 (approved 10 November 2022). No additional regulatory permits were required for this research.

### 2.2. Immunohistochemistry Marker Validation

To characterize jird peritoneal macrophages and evaluate candidate immunophenotyping reagents, PECs were harvested from naïve jirds following humane euthanasia (carbon dioxide asphyxiation with confirmatory cervical dislocation) via peritoneal lavage. Approximately 32 mL of Dulbecco’s phosphate-buffered saline (DPBS) was instilled through a 5-mm abdominal incision, and the lavage fluid was collected and pooled across animals. Cells were enumerated by hemocytometer and adjusted to 2 × 10^6^ cells/mL in DPBS. Fifty-microliter aliquots were distributed onto multichambered slides and allowed to adhere for one hour at 37 °C prior to staining. Cell pooling was used exclusively for this initial immunophenotypic characterization; all subsequent experimental animals were processed and analyzed individually.

Experimental conditions included: (1) heat-inactivated cells (65 °C, 5 min) and (2) cells maintained on ice as negative and positive viability controls, respectively; (3) cells receiving primary antibody alone; (4) cells receiving secondary antibody alone; and (5) cells receiving both primary and secondary antibodies.

The macrophage marker ionized calcium-binding adaptor molecule 1 (Iba1) was evaluated as a candidate reagent [25,27]. Rabbit anti-Iba1 primary antibody (1 µg/mL; Fujifilm Wako Pure Chemical Corporation, Osaka, Japan) was applied to adherent cells for one hour at room temperature. Following three phosphate-buffered saline (PBS) washes, a goat anti-rabbit IgG secondary antibody conjugated to Alexa Fluor™ 647 (1 µg/mL; Invitrogen, Waltham, MA, USA) was applied for 30 min at room temperature in the dark. Nuclei were counterstained with 4′,6-diamidino-2-phenylindole (DAPI), and slides were examined by fluorescence microscopy.

### 2.3. F4/80 Macrophage Marker Validation

Because commercially available jird-specific antibody reagents did not exist at the time these studies were performed, the cross-reactivity of a rat anti-mouse Alexa Fluor™ 647-conjugated anti-F4/80 monoclonal antibody (BioLegend, San Diego, CA, USA) with jird PECs was evaluated [26]. PECs were isolated and enumerated as described above. Non-specific binding was mitigated by pre-incubating cells in a blocking buffer of 2% bovine serum albumin (BSA) in Hank’s Balanced Salt Solution (HBSS; 100 µL). The anti-F4/80 antibody was added at a final concentration of 0.2 µg/mL and incubated with cells for 30 min at 4 °C. Cell viability was concurrently assessed using the LIVE/DEAD™ Fixable Violet Dead Cell Stain Kit (Thermo Fisher, Waltham, MA, USA; 1:500 dilution) per the manufacturer’s instructions. Immunostained samples were subsequently analyzed by conventional flow cytometry (NovoCyte Quanteon; Agilent, Santa Clara, CA, USA) and imaging flow cytometry (ImageStream^®^X Mk II; Luminex, Austin, TX, USA) [28].

### 2.4. Clodronate and PBS Liposomes

No established protocol for clodronate liposome (CL) administration in the jird existed at the time of study design; accordingly, a preliminary validation study was performed to determine an effective dose and regimen [26]. Clodronate liposomes and PBS liposomes were purchased from Liposoma (catalog no. CP-030-030; Amsterdam, The Netherlands). Eight jirds (*n* = 3, CL liposomes; *n* = 1, PBS liposomes) received IP injections of liposomes at 100 µL per 10 g body weight (BW) on days −4 and −1 relative to scheduled euthanasia. PECs were collected by peritoneal lavage on the day of euthanasia as described above. Cells from each animal were individually immunostained with the validated anti-F4/80 reagent and analyzed by standard flow cytometry to confirm macrophage depletion efficiency prior to initiating the infection study.

### 2.5. Parasites

*Dirofilaria immitis* L3 were obtained from laboratory-colonized *Aedes aegypti* (black-eyed Liverpool strain) mosquitoes [29] that had been allowed to feed on a microfilaremic dog maintained at the University of Georgia [30]. Larvae were recovered by dissection of the head and mouthparts of each mosquito 15 days after the infectious blood meal, following established filariasis reagent resource protocols [31]. Harvested L3 were maintained in RPMI-1640 medium supplemented with 10% fetal bovine serum at 37 °C under 5% CO_2_ until inoculation.

### 2.6. Peritoneal Macrophage Depletion and Infection with D. immitis

**Figure 1:**
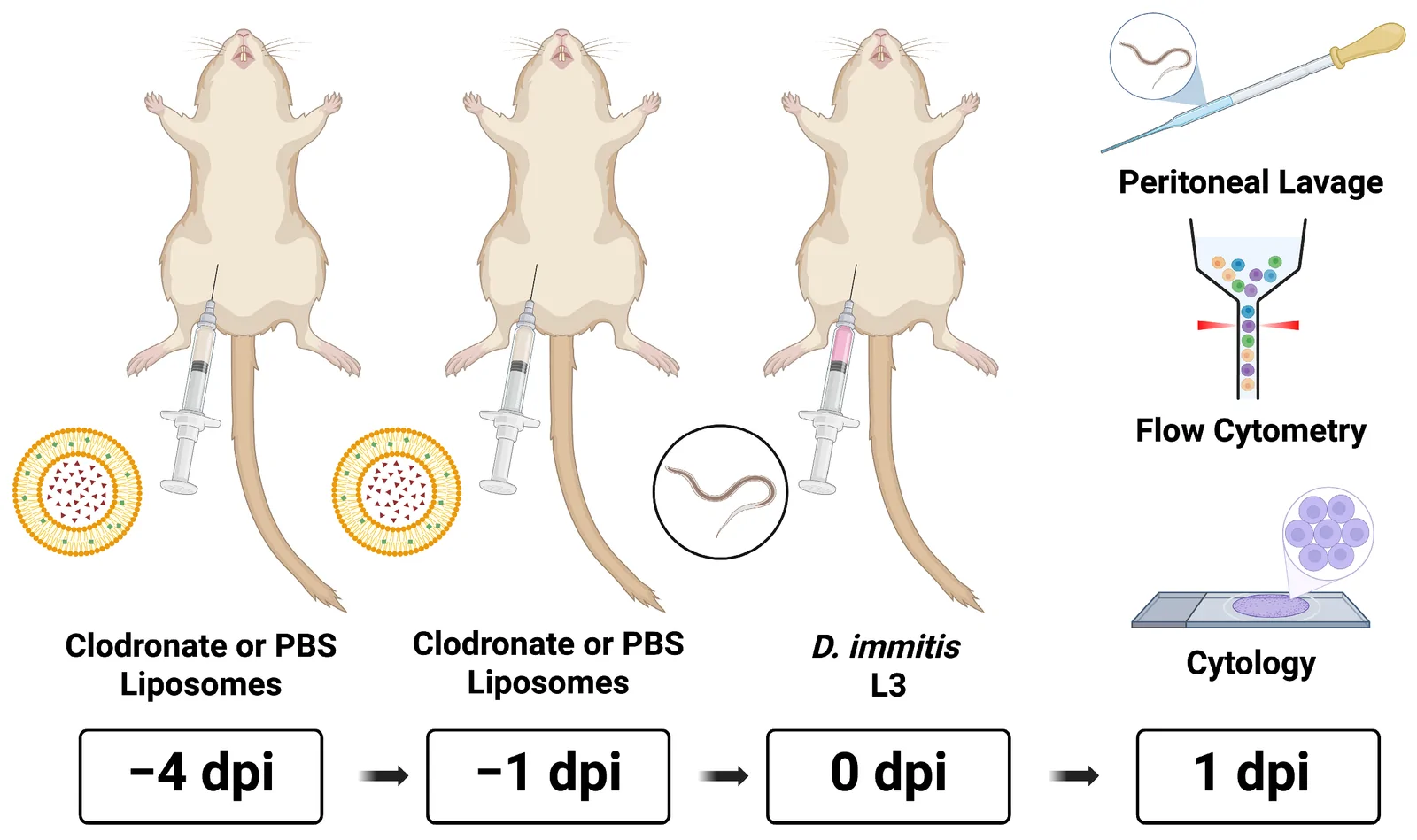
Experimental design for the depletion of macrophages in the jird model. Adult male jirds were administered clodronate (CL) or phosphate-buffered saline (PBS) liposomes (100 µL/10 g BW) via intraperitoneal injection 4 and 1 day(s) prior to infection with 40 *Dirofilaria immitis* L3. Peritoneal exudative cells (PECs) and larvae were recovered by peritoneal lavage 1-day post-infection (dpi). The PECs were utilized for macrophage depletion confirmation via flow cytometry and cytology. BW, body weight; L3, third-stage larvae. Created with BioRender.com.

For the primary experiment, jirds were randomly assigned to one of two treatment groups: peritoneal macrophage depletion (CL liposomes; *n* = 6) or vehicle control (PBS liposomes; *n* = 6). Liposomes were delivered by IP injection at 100 µL/10 g BW on days −4 and −1 relative to infection, consistent with the validated depletion regimen. On the day of infection, each jird received an IP inoculum of approximately 40 *D. immitis* L3 (Figure 1).

### 2.7. Recovery of L3 and PECs

Animals were humanely euthanized at 1 dpi by carbon dioxide asphyxiation followed by confirmatory cervical dislocation. Peritoneal contents were recovered by lavage with approximately 32 mL of DPBS instilled through a 5-mm abdominal incision. Lavage fluid was examined systematically under a dissection stereomicroscope, and all recovered L3 were enumerated. Larval recovery was expressed as a percentage of the original inoculum (number recovered ÷ number inoculated × 100).

### 2.8. Cytology

**Figure 2:**
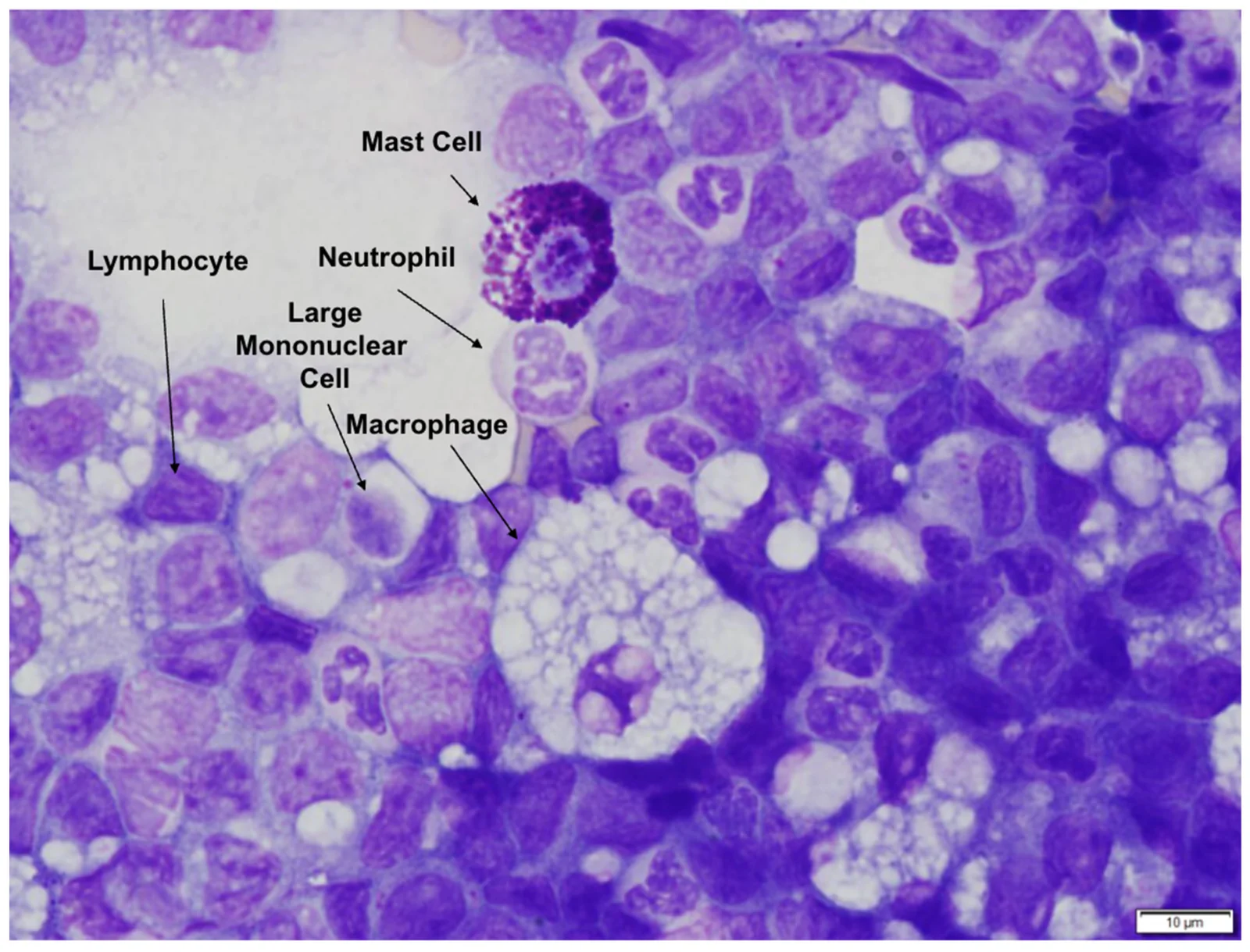
Jird peritoneal exudate cells (PECs) at 1 day post infection (dpi). Representative image of a Wright’s-stained, cytocentrifuged smear used for the 200-cell differential count. Identified cell types include lymphocytes, large mononuclear cells, macrophages, neutrophils, and mast cells. Scale bar: 10 µm.

Differential leukocyte counts were performed on cytocentrifuged preparations of peritoneal lavage cells. A 0.5-mL aliquot of cell suspension from each individual jird was deposited onto a glass slide using a Thermo Scientific™ EZ Cytofunnel™ assembly and centrifuged at 115× *g* for 5 min in a Thermo Scientific™ Cytospin 4 Cytocentrifuge. Slides were stained with a modified Romanowsky stain (Diff-Quik; Siemens Healthineers, Munich, Germany), and a blinded 200-cell differential count was conducted for each preparation to quantify the proportional representation of each peritoneal leukocyte population (Figure 2) [32].

### 2.9. Flow Cytometry

For immunophenotypic analysis of experimental PECs, cells from each individual jird were divided equally into four 12 × 75-mm polystyrene tubes containing 1 mL of DPBS, designated as: (1) unstained cells (negative control); (2) viability stain only; (3) anti-F4/80 antibody only; and (4) combined viability stain and anti-F4/80 antibody. Staining was performed at validated concentrations (LIVE/DEAD™ Fixable Violet Dead Cell Stain, 1:500; anti-F4/80, 0.2 µg/mL). All samples were acquired on the NovoCyte Quanteon flow cytometer using instrument settings standardized during the validation study.

### 2.10. Data Analysis

Analysis of flow cytometry was conducted in FlowJo™ Software v10.8 (Becton, Dickinson and Company, Ashland, OR, USA). Distribution normality for larval recovery and flow cytometry outcome variables was confirmed prior to group comparisons. Normally distributed data were analyzed using one-tailed, unpaired Welch’s *t* tests. Cytology data exhibited a non-normal distribution and were analyzed using one-tailed Mann–Whitney U tests. All statistical analyses were performed in GraphPad Prism 10 (GraphPad Software, San Diego, CA, USA), with a significance threshold set at *p* < 0.05.

## 3. Results

### 3.1. Cross-Reactive Macrophage Marker

**Figure 3:**
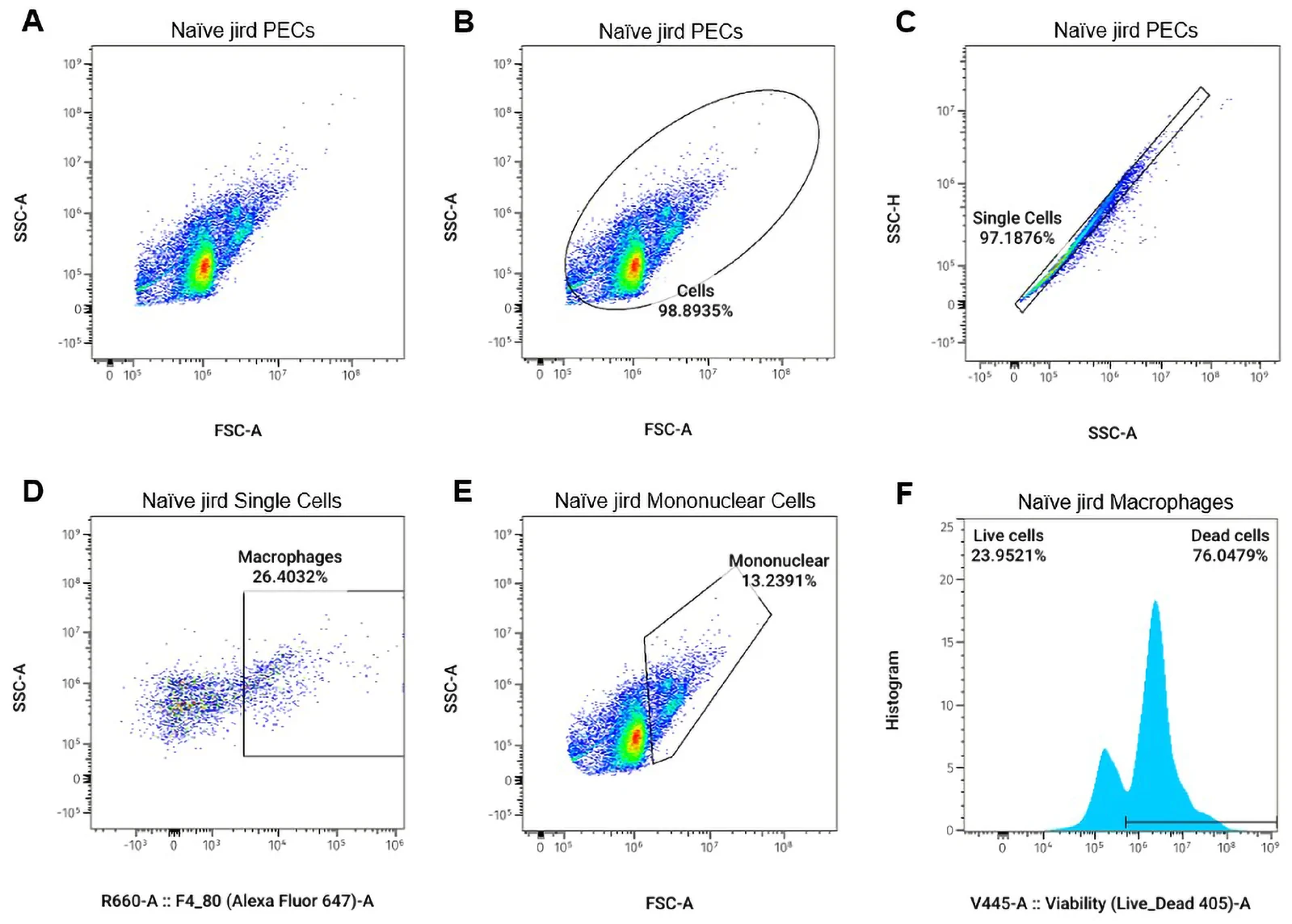
Gating strategy for the validation of cell viability stain and macrophage marker. Peritoneal exudate cells (PECs) from a naïve jird were analyzed to confirm antibody cross-reactivity. (**A**) Total PECs displayed on a dot plot of forward light scatter (FSC-A) versus side light scatter (SSC-A). (**B**) Exclusion of debris from the cell population. (**C**) Gating of single cells (SSC-H vs. SSC-A) to exclude doublets. (**D**) Identification of the mononuclear cell population (13.3% of single cells). (**E**) Quantification of macrophages (26.0% of mononuclear cells) based on F4/80 fluorescent emission (Alexa Fluor 647). (**F**) Histogram of the macrophage population gated for viability; the right peak indicates dead cells (76.6%), and the left peak indicates live cells (23.4%) based on the fluorescent emission of the violet viability stain.

The rat anti-mouse Alexa Fluor™ 647-conjugated anti-F4/80 monoclonal antibody demonstrated cross-reactivity with jird PECs, and the LIVE/DEAD™ Fixable Violet Dead Cell Stain Kit was similarly confirmed as applicable to jird cells (Figure 3). Viable macrophages from naïve jirds exhibited the expected immunophenotype: F4/80-positive and LIVE/DEAD™-negative. Under the staining conditions evaluated, Iba1 immunostaining did not produce differential labeling between macrophages and other cell types within the PEC population and was therefore excluded from subsequent experimental protocols.

### 3.2. Clodronate and PBS Liposome Validation

**Figure 4:**
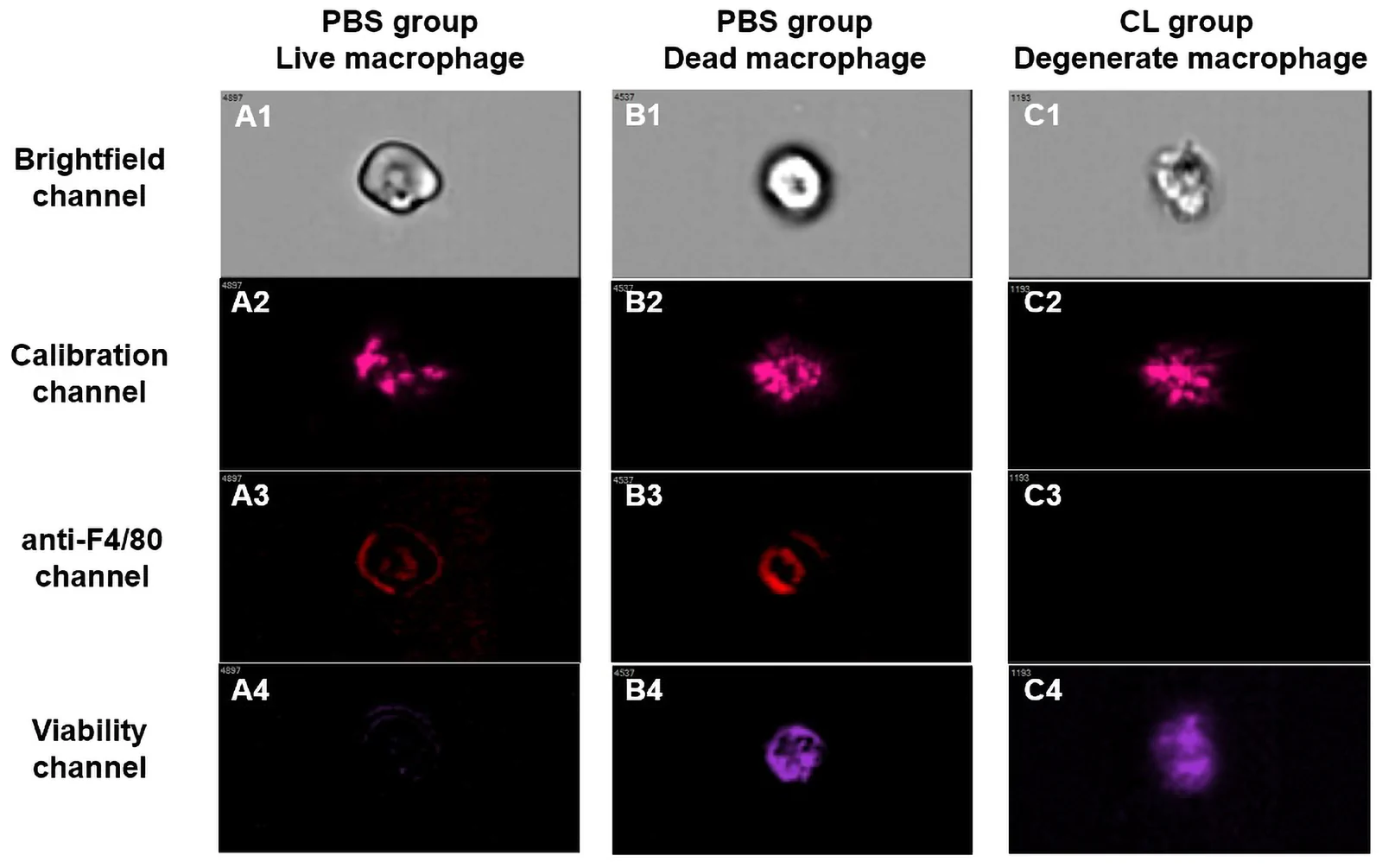
Imaging flow cytometry confirmed F4/80 cross-reactivity with jird macrophages. PECs were recovered from jirds 1 dpi and analyzed via the ImageStream^®^X Mk II. (**A1**–**A4**) A live, F4/80+ macrophage from a jird administered PBS liposomes displaying a clear cell wall. (**B1**–**B4**) A dead, F4/80+ macrophage from a jird administered PBS liposomes displaying early degradation of the cell wall. (**C1**–**C4**) A dead, F4/80- macrophage from a jird administered CL liposomes displaying signs of cell death. Row 1 (**A1**,**B1**,**C1**) images in the brightfield channel were used to assess cell morphology and exclude doublets. Row 2 (**A2**,**B2**,**C2**) displays the calibration channel with speed beads for internal calibration. Row 3 (**A3**,**B3**,**C3**) shows the anti-F4/80 channel (red). Row 4 (**A4**,**B4**,**C4**) shows the viability channel (purple) indicating dead cells.

**Figure 5:**
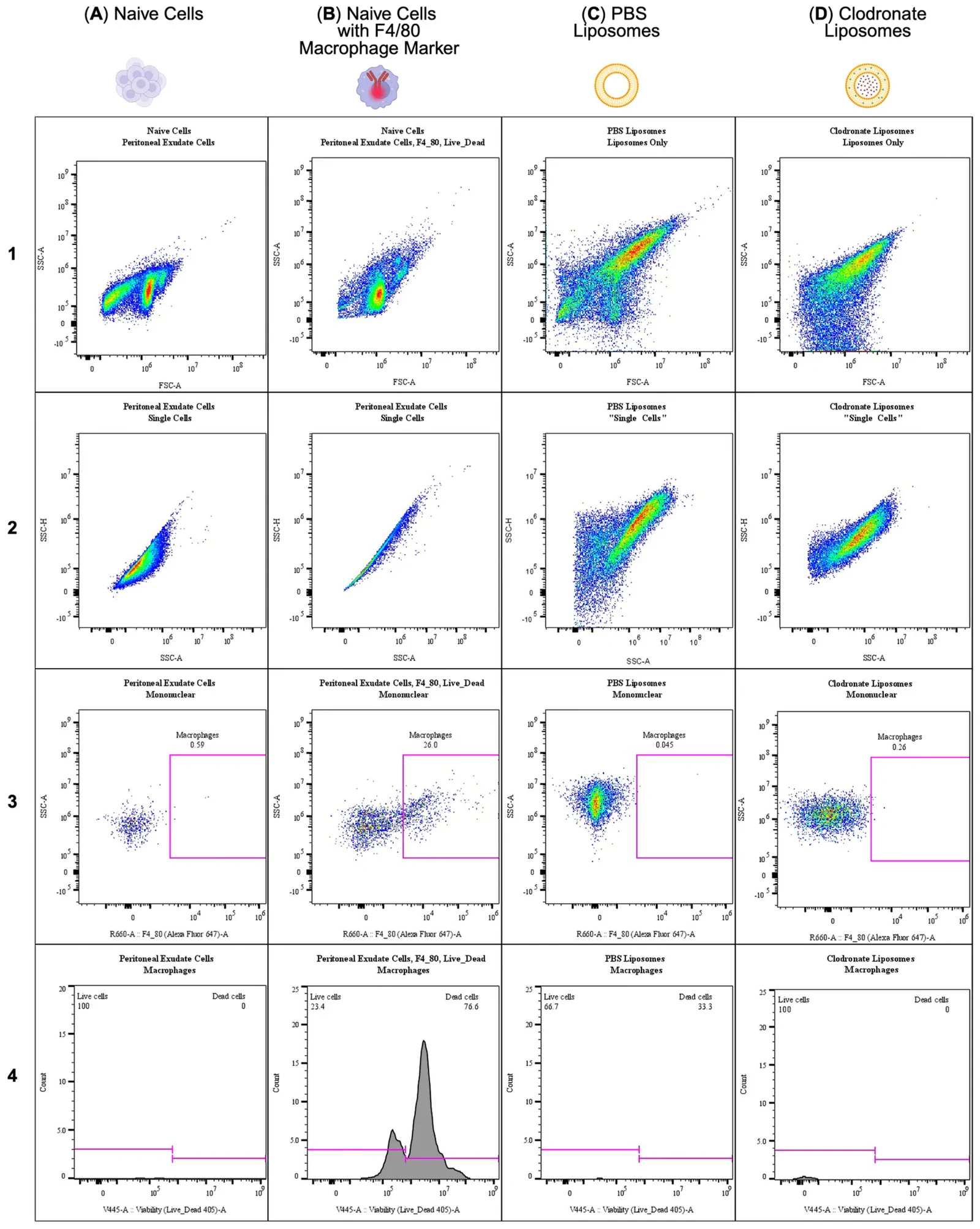
Gating strategy for the validation of macrophage depletion via flow cytometry. Peritoneal exudate cells (PECs) were recovered by peritoneal lavage and analyzed using the NovoCyte Quanteon and FlowJo Software. Row 1 (**A1**–**D1**) PECs displayed as a dot plot (FSC-A vs. SSC-A) with debris excluded. Row 2 (**A2**–**D2**) Single-cell gating (SSC-H vs. SSC-A) to exclude doublets. Row 3 (**A3**–**D3**) Macrophage gating within the mononuclear cell population according to F4/80 fluorescent emission (Alexa Fluor 647). Row 4 (**A4**–**D4**) Histograms displaying live and dead cells gated by the fluorescent emission of the viability stain. Column (**A**) Naïve PECs without F4/80 or viability stain. Column (**B**) Naïve PECs with F4/80 and viability stain. Column (**C**) Control group administered PBS liposomes. Column (**D**) Macrophage-depleted group administered CL liposomes.

The CL dose and administration regimen were validated by imaging and standard flow cytometry prior to the infection experiment. Imaging flow cytometry confirmed F4/80 cross-reactivity with jird macrophages in naïve animals and those inoculated with larvae, with live F4/80+ macrophages clearly identifiable in both populations (Figure 4). Standard flow cytometry provided complementary confirmation of macrophage depletion efficacy (Figure 5): PBS liposome-treated controls and naïve samples displayed a well-defined macrophage population within the monocyte gate (characterized by intermediate side scatter and high F4/80 expression), whereas this population was markedly diminished or absent in CL-treated samples.

### 3.3. Larval Recovery

**Figure 6:**
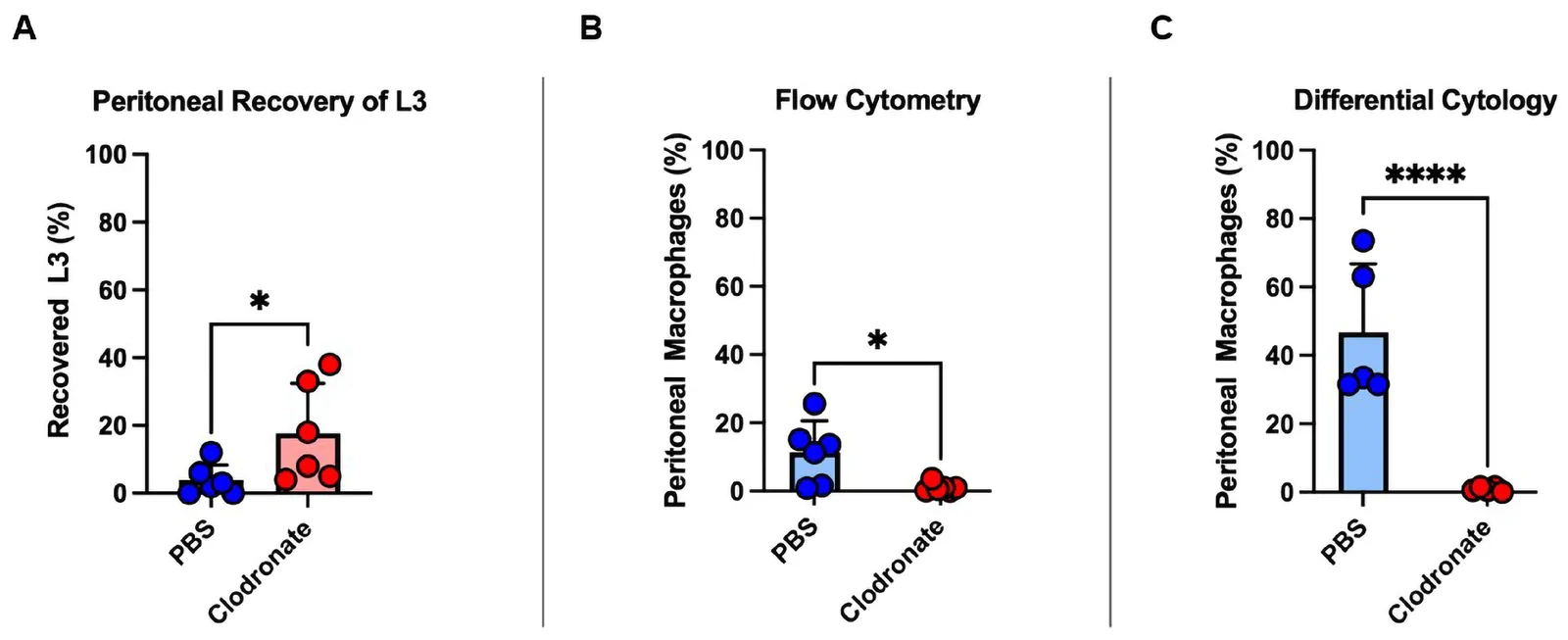
Recovery of larvae and macrophage presence across experimental groups. (**A**) Significantly (* = *p* < 0.03) more *D. immitis* L3 were recovered from macrophage-depleted jirds than from control jirds at 1-day post-infection (dpi). (**B**) Flow cytometry analysis confirming significantly fewer (* = *p* < 0.02) peritoneal macrophages in the CL-treated group. (**C**) Cytologic analysis confirming a significant reduction (**** = *p* < 0.0001) in macrophages in the CL-treated group. CL, clodronate; L3, third-stage larvae; SEM, standard error of the mean.

**Figure 7:**
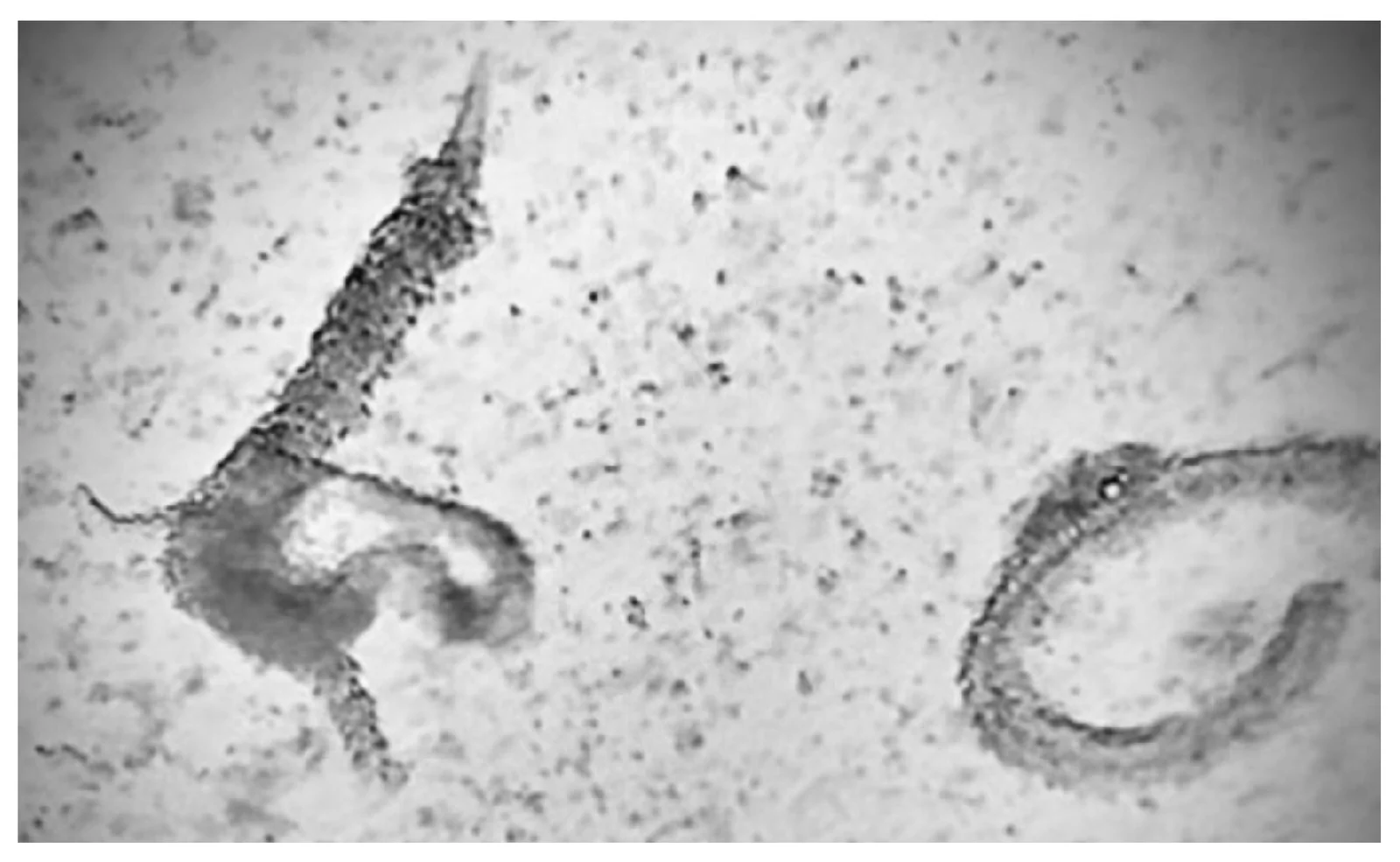
Cell attachment to *D. immitis* L3 in a control jird. PECs observed encapsulating one (**left**) of two (**right**) larvae recovered from the peritoneal cavity of a jird administered PBS liposomes.

**Figure 8:**
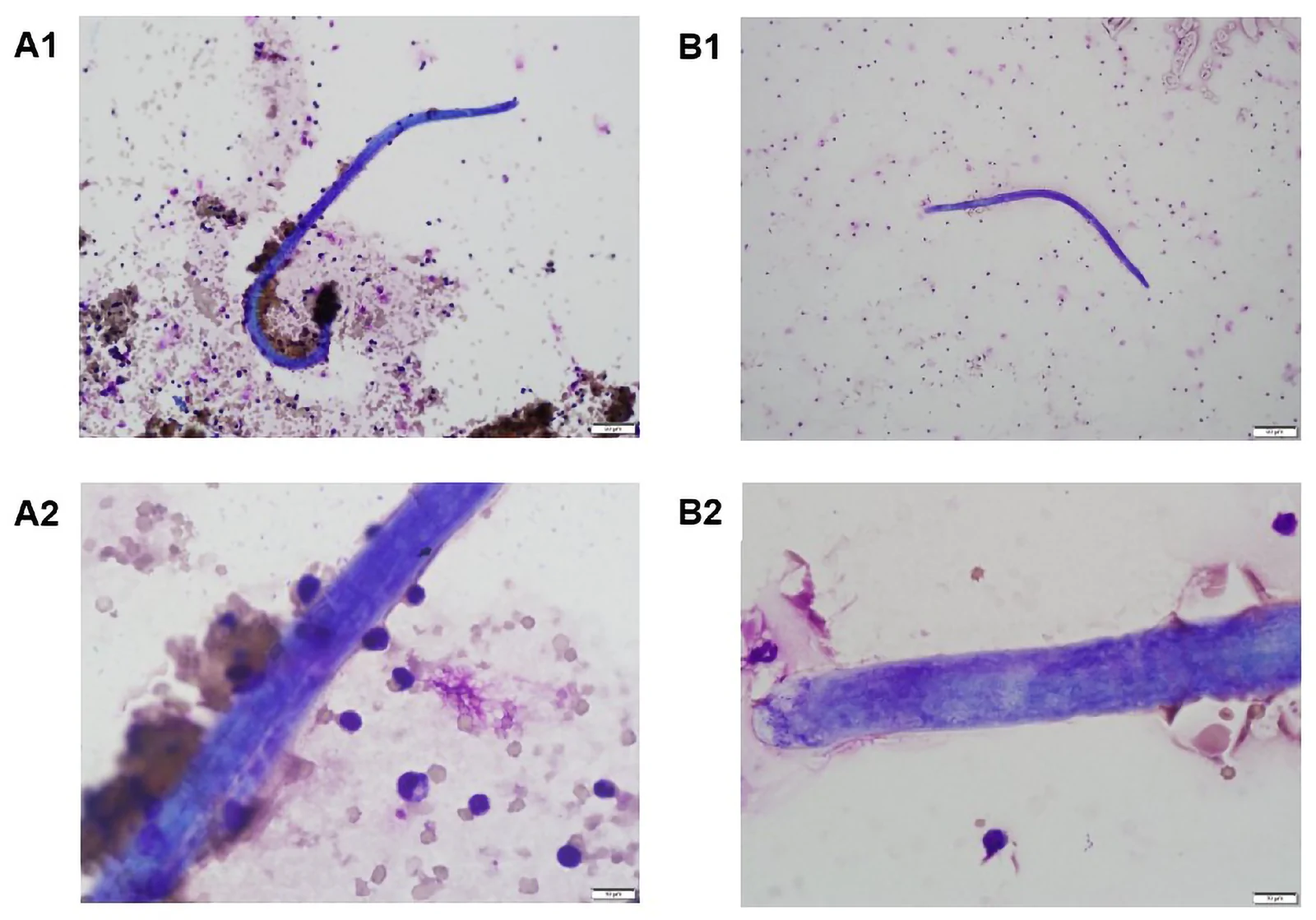
Cell attachment to *D. immitis* L3 in a macrophage-depleted jird. (**A1**,**A2**) PECs attached to an L3; however, the cells are not characterized as activated macrophages based on their small morphology. (**B1**,**B2**) Example of an L3 with no cell attachment. PECs, peritoneal exudate cells.

Significantly greater numbers of *D. immitis* L3 were recovered from macrophage-depleted jirds (mean = 17.7%; SEM = 6.0%) than from PBS liposome-treated controls (mean = 3.8%; SEM = 1.9%) at 1 dpi (*t*(6) = 2.20, *p* < 0.03; Figure 6A). Morphologically, larvae recovered from macrophage-depleted animals were predominantly unencapsulated or only loosely associated with peritoneal cells, whereas larvae recovered from control jirds were typically encapsulated by large, morphologically activated macrophages. Cell attachment to larvae was observed in two of six control jirds (Figure 7) and one of six macrophage-depleted jirds (Figure 8).

### 3.4. Flow Cytometry

**Figure 9:**
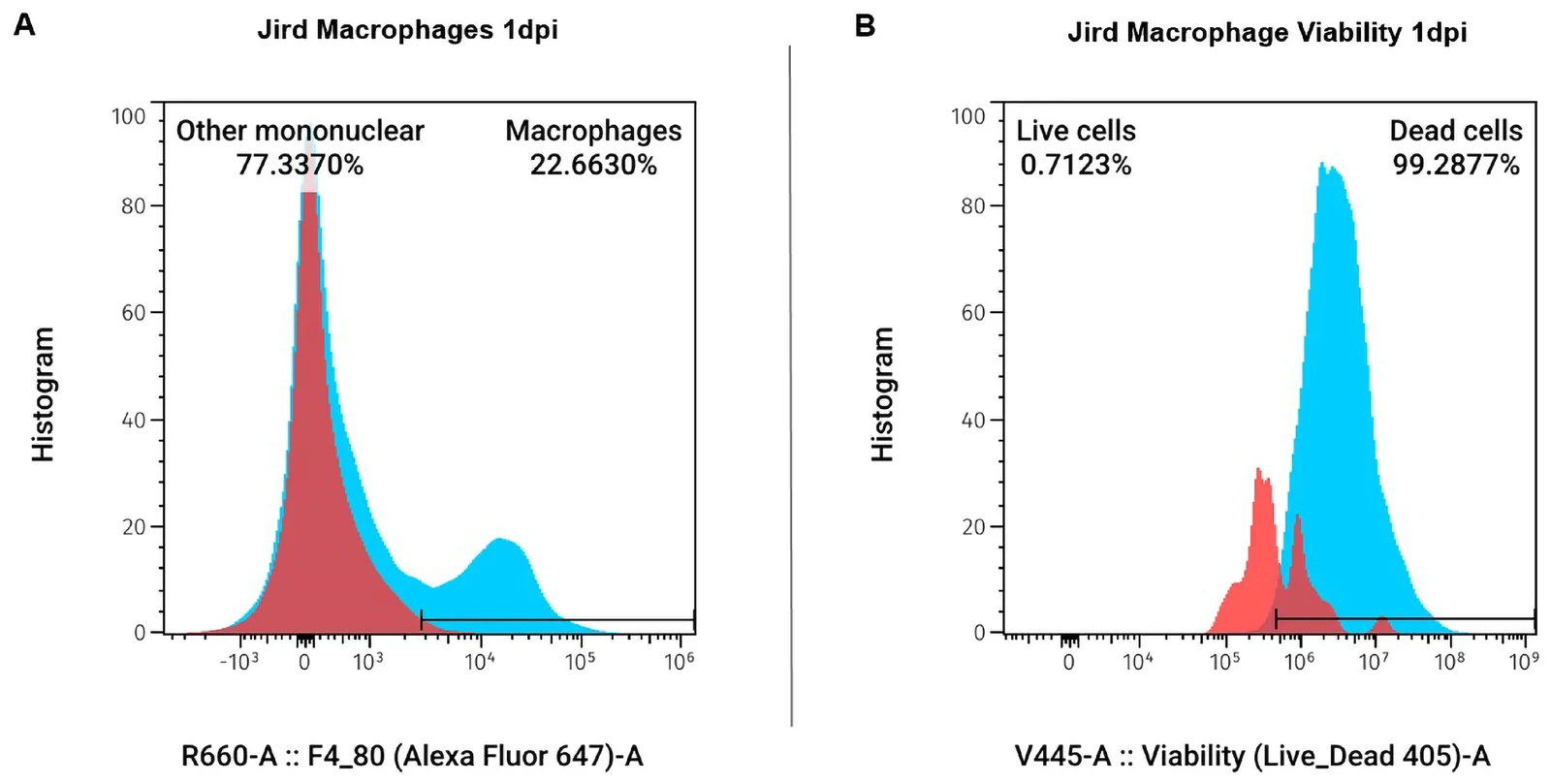
Comparative histogram of macrophage presence. (**A**) Histogram showing F4/80 fluorescence in a CL-treated sample (red) versus a PBS control (blue). (**B**) Histogram displaying live and dead macrophages gated by viability stain emission in a CL-treated sample (red) versus a PBS control (blue).

**Figure 10:**
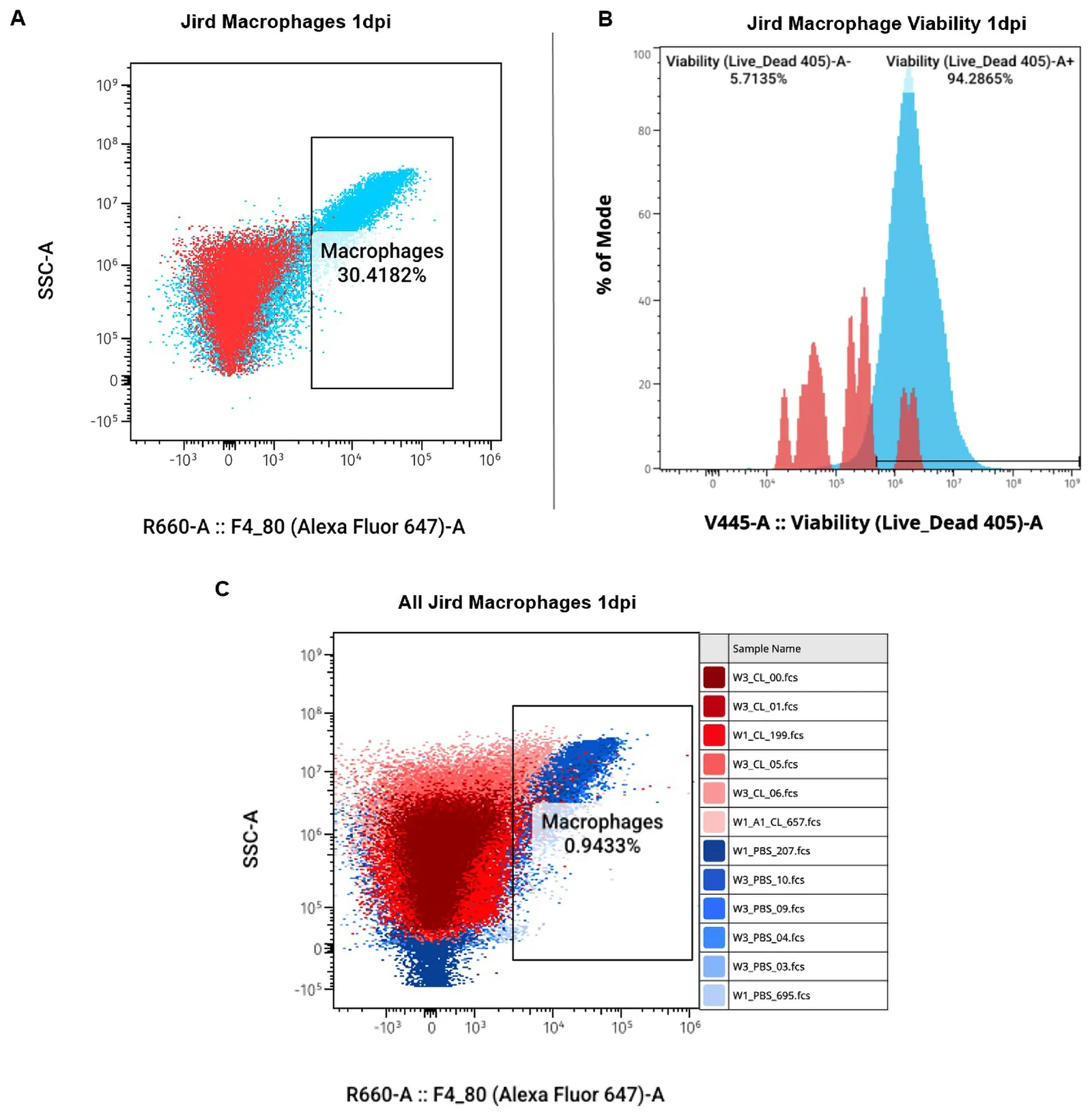
Combined flow cytometry data for all infected jirds. (**A**): Dot plot (F4/80 vs. SSC-A) for a representative CL-treated and PBS-treated sample depicting the decrease of macrophages recovered from CL-treated jirds 1 dpi. (**B**): There were very few viable macrophages recovered from the CL-treated jirds as indicated by the viability histogram for the same samples in (**A**). (**C**): An overlaid dot plots for all samples are depicted to represent the number of macrophages recovered from CL-treated fewer than PBS-treated jirds (red: CL group; blue: PBS group).

Standard flow cytometric analysis of PECs at 1 dpi revealed a significantly lower proportion of peritoneal macrophages in CL-treated jirds (mean = 1.1% of total PECs; SEM = 0.5%) compared with PBS liposome-treated controls (mean = 11.6% of total PECs; SEM = 2.5%) (*t*(5) = 3.95, *p* < 0.006; Figure 6B). Visual representations of these differences across individual animals are shown in Figure 9 and Figure 10.

### 3.5. Cytology

**Figure 11:**
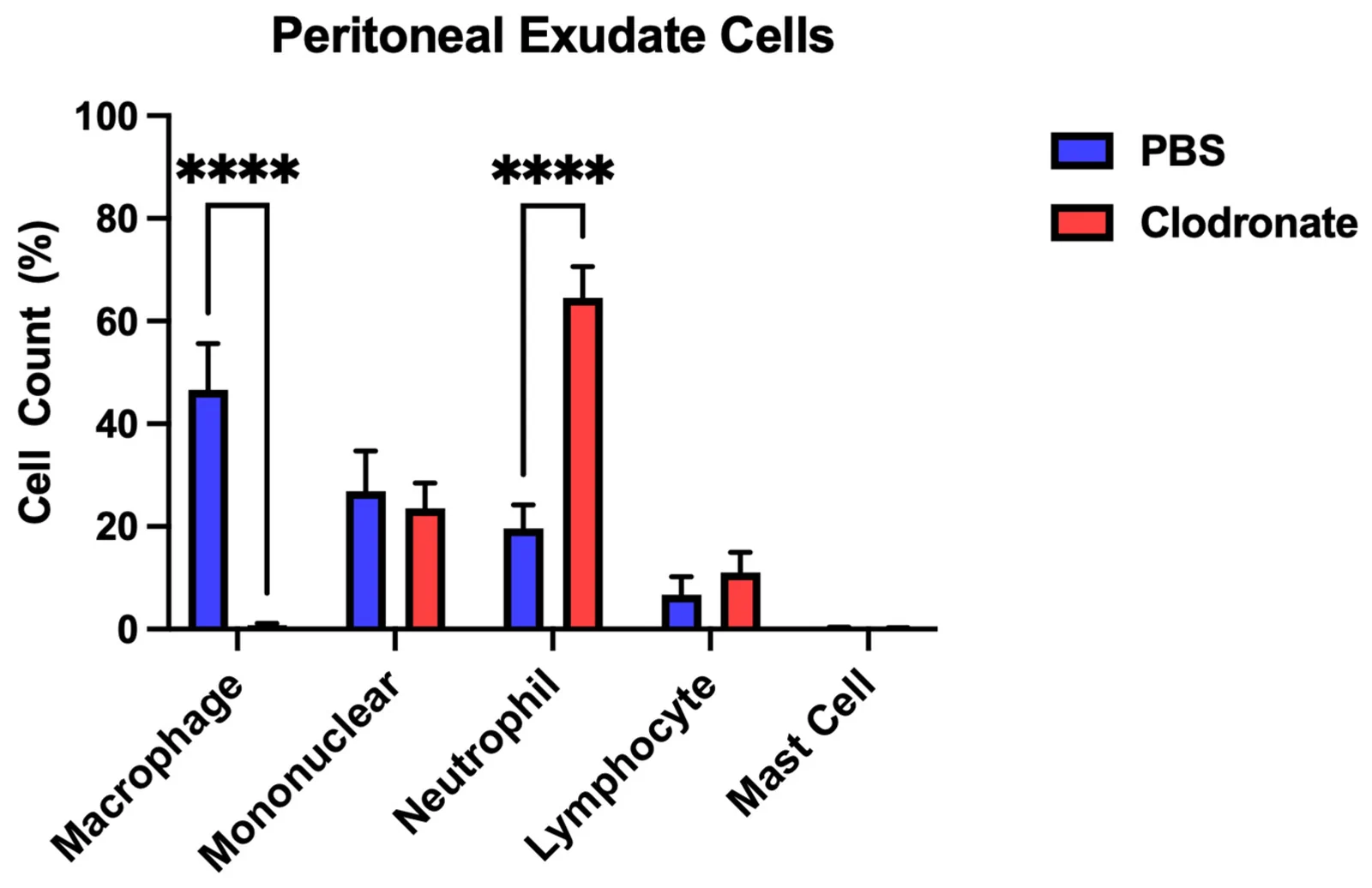
Percentage of peritoneal exudate cell types by cytology. Macrophage-depleted jirds (red) showed a significant (*p* < 0.0001) decrease in macrophages and a significant (*p* < 0.0001) increase in neutrophils compared to PBS controls (blue). No significant differences were observed for other cell types. Asterisks (****) indicate statistical significance (*p* < 0.0001).

**Figure 12:**
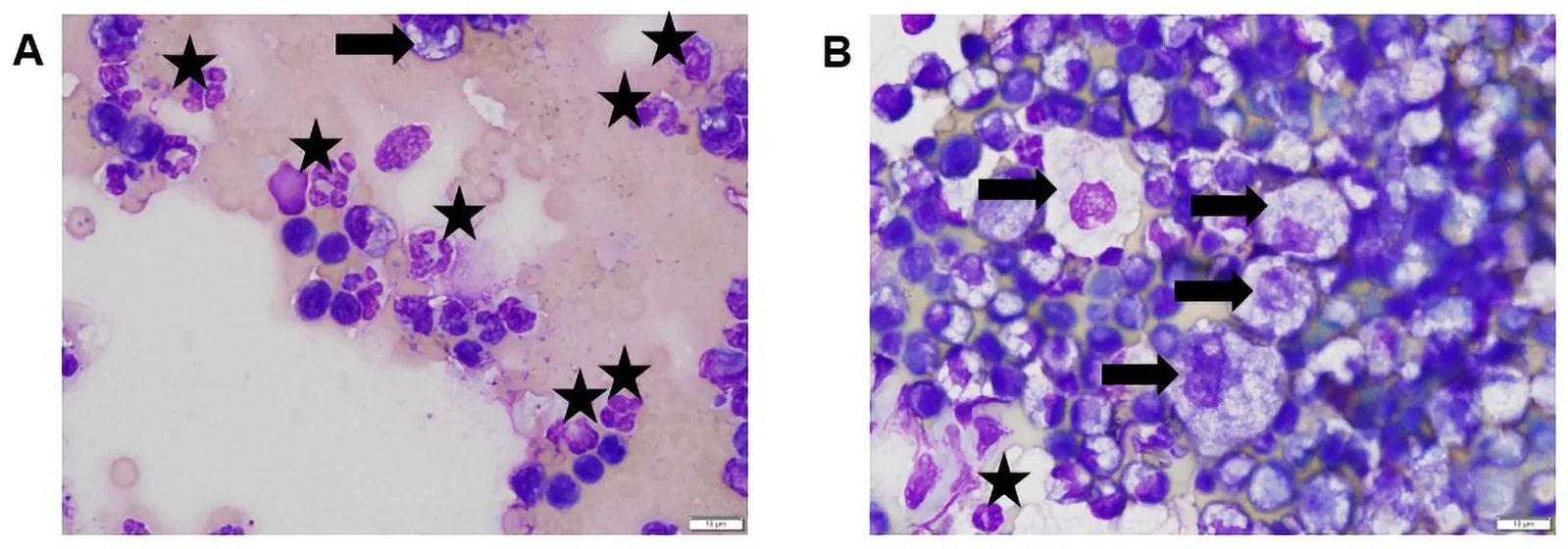
Cytologic comparison of peritoneal populations. (**A**) Representative image from a macrophage-depleted jird showing a predominance of neutrophils (stars) and a lack of macrophages. (**B**) Representative image from a control jird showing prominent macrophages (arrows) and fewer neutrophils.

Differential cell counts from cytocentrifuged peritoneal preparations revealed significant between-group differences in the proportional representation of individual leukocyte populations (Figure 6C, Figure 11). Macrophages were profoundly depleted in CL-treated jirds (mean = 0.8%; SEM = 0.3%) relative to PBS liposome controls (mean = 46.6%; SEM = 6.4%) (*t*(5) = 7.22, *p* < 0.0001). Concomitant with macrophage depletion, a significant compensatory increase in neutrophils was observed: CL-treated jirds exhibited a markedly higher neutrophil fraction (mean = 64.5%; SEM = 5.7%) compared with controls (mean = 19.6%; SEM = 5.1%) (*t*(9) = 5.86, *p* < 0.0001; Figure 11 and Figure 12). No statistically significant between-group differences were detected for lymphocytes (*p* = 0.42), mast cells (*p* > 0.99), or large mononuclear cells (*p* = 0.72). The large mononuclear cell fraction may represent peripherally derived monocytes recruited to the peritoneal compartment as a secondary response to macrophage depletion.

## 4. Discussion

The principal finding of this study, that peritoneal macrophage depletion significantly elevated the recovery of *D. immitis* L3 from jird peritoneal cavities at 1 dpi (*p* < 0.03; Figure 6A), provides direct experimental support for the hypothesis that macrophages are central to the innate cellular defense against filarial larval establishment in a nonpermissive host. The marked contrast in larval recovery between macrophage-depleted jirds (mean = 17.7%) and PBS liposome controls (mean = 3.8%) indicates that peritoneal macrophages are responsible, either through direct effector activity or through coordination of downstream immune cell populations, for the sequestration or elimination of L3 from the free peritoneal space following inoculation.

Access to *D. immitis* for experimental use is constrained in most research settings, a logistical reality that has historically driven the field toward in vitro approaches using microfilariae and L3 [33,34,35]. In vitro co-culture systems have demonstrated that macrophages can associate with and kill *D. immitis* L3 [33,34,35]; however, the extent to which these observations reflect the in vivo peritoneal environment has remained uncertain. The tractable nonpermissive jird model validated here offers a complementary in vivo platform for interrogating innate cellular responses to *D. immitis* L3 without requiring access to permissive definitive hosts.

The selective depletion of macrophages using clodronate liposomes exploits a well-characterized intracellular mechanism. Free clodronate is membrane-impermeant and rapidly cleared renally when administered in unencapsulated form [36,37]. Encapsulation within phosphatidylcholine, cholesterol liposomes render it a substrate for macrophage phagocytosis; intracellular degradation of the lipid bilayer releases clodronate into the cytosol, where accumulation progressively disrupts mitochondrial membrane function and induces macrophage-selective apoptosis [38].

No species-specific CL protocol existed for the jird at the time of study initiation; accordingly, the depletion regimen was adapted from published Wistar rat data [39], which demonstrated effective peritoneal macrophage depletion with 100 µL/10 g BW administered over a 3–4-day window. The adapted regimen, IP injections on days −4 and −1, was prospectively confirmed by flow cytometry to produce a significant reduction in F4/80+ peritoneal cells in CL-treated animals relative to PBS controls. This validation step was essential to ensure interpretive confidence in larval recovery differences observed between experimental groups. The use of PBS liposomes as the vehicle control, rather than untreated naïve jirds, was designed to account for any non-specific inflammatory effects of the liposome carrier itself. Resource constraints precluded inclusion of a third, non-injected naïve group within this experimental design.

Immunophenotypic characterization of jird peritoneal macrophages required a cross-species reagent approach owing to the absence of commercially available jird-specific antibodies. The rat anti-mouse Alexa Fluor™ 647-conjugated anti-F4/80 monoclonal antibody was confirmed to bind selectively to a population of jird peritoneal cells with morphological and phenotypic features consistent with macrophages. To our knowledge, this is the first application of imaging flow cytometry to macrophage identification in the jird, providing single-cell morphological data sufficient to corroborate the F4/80-based immunophenotypic assignment. It should be noted, however, that formal molecular characterization of macrophage polarization state during *D. immitis* challenge, such as arginase-1 pathway activation, was not performed in the present study and constitutes a recognized limitation.

The larval recovery rate in PBS control jirds in the present study (mean = 3.8%) was modestly higher than the 0.4% reported by Campbell et al. [22]. This inter-experiment discrepancy is most plausibly attributable to biological variability in host immune competence across individual animals and to differences in L3 fitness across parasite preparations [22]. Because the two studies used independent animal cohorts and parasite pools, within-study treatment comparisons remain the appropriate unit of inference.

A secondary finding of considerable mechanistic interest was the significant compensatory expansion of the peritoneal neutrophil population in macrophage-depleted jirds (mean = 64.5%) relative to controls (mean = 19.6%; *p* < 0.0001). Neutrophils constitute the earliest-responding circulating innate effectors, mobilized to sites of infection by chemoattractant signals ordinarily released in part by macrophages and tissue-resident cells [35]. The robust neutrophil influx in depleted animals demonstrates that the peritoneal immune compartment mounts a compensatory cellular response in the absence of macrophages. However, the concurrent increase in larval recovery from these animals indicates that neutrophils alone cannot replicate macrophage-mediated larval clearance, identifying macrophages as the non-redundant primary effector in this host–parasite interaction.

Cytologic examination of PBS control jirds revealed large, morphologically activated macrophages (exceeding 50 µm) in direct contact with larvae at the peritoneal wall and diaphragm, consistent with active larval encapsulation. Helminth-driven Th2 cytokine responses mediated through interleukin (IL)-4 and IL-13 classically polarize tissue macrophages toward an alternatively activated (M2/AAM) phenotype, promoting tissue remodeling and parasite encapsulation over canonical pro-inflammatory killing [9,10]. AAM involvement in host responses to filarial nematodes has been documented across multiple experimental systems [11,12], and the morphological evidence presented here is consistent with AAM-driven larval sequestration into peritoneal wall and diaphragmatic tissue in the nonpermissive jird host.

The central role of macrophages in determining larval establishment in the present study is consistent with their recognized importance across experimental filarial models, though the directionality of macrophage effects varies by host-parasite combination. In the *Litomosoides sigmodontis* rodent model of filariasis, alternatively activated macrophages have been identified as central mediators of larval development and establishment. In the permissive host, Th2-driven arginase pathway polarization by AAMs promotes larval establishment rather than clearance [11]. Divergent macrophage roles, promoting either parasite clearance or immune tolerance, have similarly been described in *Brugia*-based models of lymphatic filariasis [12]. Neutrophils have also been identified as secondary innate effectors across multiple filarial systems yet are insufficient to compensate for macrophage-mediated clearance when macrophage populations are depleted or exhausted [35]. The present results in the jird *D. immitis* model are broadly consistent with this emerging consensus across filarial parasite-host systems.

A recognized limitation of the present experimental design is the use of intraperitoneal inoculation as the route of infection, which does not replicate natural mosquito-mediated percutaneous deposition of L3. Intraperitoneal challenge was selected because it permits controlled, quantitative larval delivery and efficient PEC recovery by peritoneal lavage. Whether the macrophage-dependent clearance mechanism characterized here operates equivalently in the subcutaneous compartment following natural mosquito challenge remains an open question. Studies employing natural-route infection would be required to determine the extent to which these IP inoculation-based findings translate to vector-borne transmission conditions.

Definitive biochemical confirmation that *D. immitis* L3 actively drive AAM polarization in the jird will require demonstration of arginase-1 pathway activation, a canonical molecular hallmark of M2 polarization [40,41]. Arginase activity assays on peritoneal macrophages from infected jirds represent a directly accessible next step and would provide the molecular evidence needed to connect larval immune evasion signaling to the observed macrophage effector phenotype. Elucidating these signaling events may identify pharmacologically targetable nodes at the host–parasite interface with relevance to prevention and treatment of heartworm disease and related neglected filarial infections [26].

## 5. Conclusions

Selective depletion of peritoneal macrophages led to significantly greater recovery of *D. immitis* L3 from jird peritoneal cavities at 1 dpi, providing direct experimental evidence that macrophages are the primary innate cellular mediators of larval clearance in this nonpermissive host. In their absence, a compensatory but insufficient neutrophil response emerged, highlighting the non-redundant role of macrophages in this host–parasite interaction. These findings establish a validated in vivo experimental framework, including a cross-reactive F4/80 flow cytometric reagent and an efficacious IP clodronate liposome depletion protocol, for mechanistic dissection of the cellular determinants of filarial host specificity. Future studies employing this platform to characterize macrophage activation state, peritoneal cytokine milieu, and arginase pathway engagement will further clarify the molecular signals governing *D. immitis* larval fate and may reveal intervention targets applicable to filarial disease control [26].

## Data Availability

The data presented in this study are available on request from the corresponding author.

## Abbreviations

The following abbreviations are used in this manuscript:

AAMs	Alternatively activated macrophages
BSA	Bovine serum albumin
BW	Body weight
CAMs	Classically activated macrophages
CL	Clodronate
DPBS	Dulbecco’s phosphate-buffered saline
dpi	Days post-infection
HBSS	Hank’s Balanced Salt Solution
Iba1	Ionized calcium-binding adaptor molecule
IACUC	Institutional Animal Care and Use Committee
L3	Third-stage larvae
PBS	Phosphate-buffered saline
